# Effect of flumazenil on recovery of memory following recovery of consciousness from general anesthesia with remimazolam: a randomized, open-label, single-center controlled trial

**DOI:** 10.1186/s40981-025-00825-5

**Published:** 2025-10-21

**Authors:** Keiko Nobukuni, Kazuhiro Shirozu, Masako Asada, Taichi Ando, Etsuko Kanna, Kotaro Kakehashi, Ryotaro Shiraki, Makoto Kubo, Ken Yamaura

**Affiliations:** 1 Operating Rooms, Kyushu University Hospital, Fukuoka, Japan; 2 Department of Anesthesiology and Critical Care Medicine, Kyushu University Hospital, Fukuoka, Japan; 3 Department of Breast Surgical Oncology, Kyushu University Hospital, Fukuoka, Japan; 4 Department of Anesthesiology and Critical Care Medicine, Graduate School of Medical Sciences, Kyushu University, Fukuoka, Japan

**Keywords:** Anesthesia, Memory, Flumazenil, RCT, Remimazolam

## Abstract

**Background:**

Recovery of consciousness from general anesthesia with remimazolam, an ultrashort-acting benzodiazepine, occurs rapidly. However, patients after recovery of consciousness from general anesthesia with remimazolam often experience periods of amnesia. Remimazolam can be antagonized by flumazenil. Therefore, we investigated the effect of flumazenil on the recovery of memory following the recovery of consciousness from general anesthesia with remimazolam.

**Methods:**

This single-center randomized controlled trial was conducted from November 2023 to July 2024. Forty-four patients undergoing breast surgery were enrolled. The patients received general anesthesia with remimazolam and remifentanil and were randomized to receive flumazenil after recovery of consciousness or not. The recovery of the memory was evaluated by showing an A4-size poster (illustration) to the patients and asking them to remember the poster every 1 h. Furthermore, the effect-site concentration of remimazolam was calculated using the Masui model.

**Results:**

All 44 patients (22 with and 22 without flumazenil) were assessed. The percentage of patients who remembered the poster 1 h after regaining consciousness was significantly higher in the flumazenil group than in the no flumazenil group (95.5 vs 40.9%; *p* < 0.001). All patients could recall the poster within 2 h postoperatively. The mean effect-site concentration of remimazolam at the time of consciousness recovery was similar between the two groups (0.31 ± 0.08 µg/mL).

**Conclusions:**

Flumazenil significantly accelerated the recovery of memory retention in patients who had recovered consciousness from general anesthesia with remimazolam. However, even without the administration of flumazenil, all patients successfully recovered their memory within 2 h after regaining consciousness.

**Trial registration:**

This clinical trial was registered at the University Hospital Medical Information Network (UMIN) Center on November 01, 2023 (UMIN-CTR: UMIN000052659).

**Supplementary Information:**

The online version contains supplementary material available at 10.1186/s40981-025-00825-5.

## Background

Remimazolam is an ultrashort-acting benzodiazepine widely used during general anesthesia and sedation due to its rapid onset and short context-sensitive half-life [1]. The availability of its antagonist, flumazenil, further supports its use in procedures requiring quick recovery, including gastrointestinal endoscopy and bronchoscopy [1–3]. While research has focused on the safety and efficacy of remimazolam in procedural sedation, many of these studies have centered on endpoints such as time to eye-opening or extubation following anesthesia [2]. However, limited attention has been given to cognitive recovery, particularly memory retention after remimazolam.

Awakening from anesthesia typically proceeds from the recovery of consciousness to the recovery of memory. Intravenous anesthetics such as propofol and midazolam have been reported to inhibit memory formation and impair memory retention [3]. The time lag between the recovery of consciousness and the return of memory is considered a significant medical issue; however, it can also lead to social problems depending on the patient’s behavior, such as disinhibition due to residual low-dose anesthesia. Therefore, understanding the duration of memory impairment after the recovery of consciousness is clinically important. This is a critical gap, as impaired memory recovery after sedation could pose risks for patients discharged shortly after procedures. Memory involves the following three processes: recognition, retention, and recall. Recognition is the initial acquisition of new information. Retention is the storage of the encoded information over time. Recall is the ability to retrieve and use the stored information when needed. In other words, even if recognition is achieved at a certain point in time, if it is not retained and recalled, it will not remain as a memory. If they could recall what they saw immediately after surgery, it means that they were sufficiently recovered from anesthesia and preserved the retention and recall memory ability during the period from recognition to recall. For instance, if patients experience delayed memory retention recovery after remimazolam, early discharge following sedation could be unsafe, necessitating extended monitoring postoperatively. Even if patients could respond to verbal stimuli after extubation, they may still experience impaired memory retention and may not remember what they were told after waking from anesthesia. Our previous studies have indicated that remimazolam, when used for anesthesia, may lead to slower recovery of memory and cognition than propofol, without inducing retrograde amnesia [4]. However, we did not investigate whether flumazenil could mitigate these effects on memory retention and recall.

Flumazenil, a benzodiazepine antagonist, is known to accelerate arousal and recovery times. To date, no studies have specifically compared the recovery times for memory retention in patients treated with remimazolam with and without flumazenil. Therefore, in this study, we aimed to evaluate the impact of flumazenil on the recovery of memory retention in patients with recovery consciousness from general anesthesia using remimazolam.

## Methods

This was an open-label, single-center, randomized clinical trial that compared memory retention recovery times in patients receiving remimazolam with or without flumazenil administration. The study was approved by the Institutional Review Board of Kyushu University School of Medicine Hospital (Chairperson Prof E. Baba, approval number: 20232013, approved on October 30, 2023) and conducted in accordance with the World Medical Association Declaration of Helsinki. All participants provided written informed consent before inclusion. The trial was registered in the UMIN Clinical Trials Registry (UMIN-CTR: UMIN000052659; November 01, 2023). This study was conducted in compliance with the Consolidated Standards of Reporting Trials guidelines.

## Participants

Female patients scheduled for breast surgery under general anesthesia, aged 20–65 years, were considered for inclusion. These criteria ensured consistency in intraoperative conditions and minimized variability in cognitive function across participants. The exclusion criteria included a history of hypersensitivity to remimazolam or propofol, allergies to egg or soybean oil, acute angle-closure glaucoma, myasthenia gravis, severe systemic disease, American Society of Anesthesiologists physical status IV, shock, or coma. One patient in the remimazolam group was taking antidepressants and sleep medication, while one in the flumazenil group was using a non-benzodiazepine sleep medication. No premedication was administered to both patients.

### Randomization and blinding

Participants were randomly assigned to either the flumazenil group or the without flumazenil group through stratified block randomization, ensuring balanced group sizes. To maintain independence, an allocation supervisor from the Data Center of the Clinical Research Promotion Department, Clinical and Translational Center, prepared the randomization table without clinical involvement. Discontinuation criteria and guidelines for monitoring adverse events have been described in detail in previous research [5].

The primary endpoint was the time to recovery of memory retention. The secondary endpoint was the frequency of analgesic or antiemetic use until 3 h post-surgery.

### Anesthesia method

Remimazolam (Mundipharma K.K., Tokyo, Japan) was administered at an initial dose of 3 mg/kg/h, then reduced to 1 mg/kg/h once the patient was confirmed to have lost consciousness. During surgery, the dosage of remimazolam was adjusted as necessary within the range of 0.5–2 mg/kg/h, guided by the Patient State Index (PSI). The target PSI for both groups was set between 25 and 50. For intraoperative analgesia, standard doses of fentanyl and remifentanil were used, with the remifentanil dose capped at 0.5 µg/kg/min. After confirming the recovery of consciousness, patients were extubated.

**Fig. 1:**
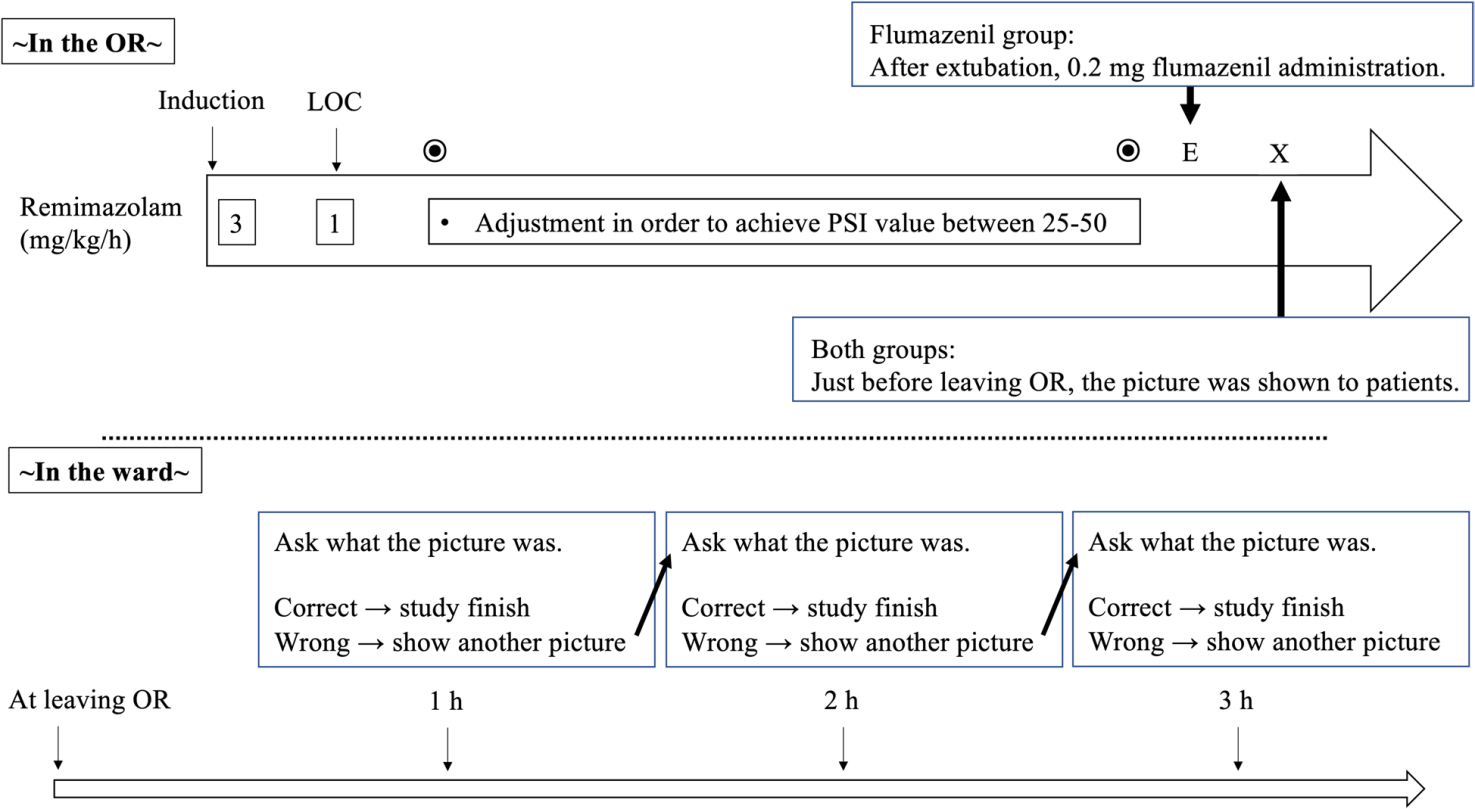
The measurement and anesthesia flow. LOC, loss of consciousness

In the flumazenil group, 0.2 mg of flumazenil was administered after the recovery of consciousness and extubation (Fig. 1).

### Measurements

Patients were shown an A4-size poster (illustration A) for a few seconds and asked to identify the items depicted once they had reached a Modified Aldrete score of ≧8 and could state their own name after extubation. Memory retention for illustration A was assessed 1 h after showing the poster. If the patient could not recall illustration A, a second poster (illustration B) was shown at that time (1 h after showing the poster (illustration A). Subsequently, memory retention for illustration B was reassessed 1 h after showing Illustration B. If the patient could not recall illustration B, a third poster (illustration C) was shown at that time (1 h after showing the poster (illustration B). A blinded investigator performed all memory evaluations. No changes were made to the trial outcomes after the trial commenced. Illustrations were laminated A4-size posters that contained easily and simply recognizable images, such as a car, a dog, and a banana (see Additional file 1).

The effect-site concentration of remimazolam was calculated using Masui et al.’s model [6, 7], which applies even when flumazenil is administered [8]. The Shafer’s pharmacokinetic model was used to predict the effect-site concentration of fentanyl [9].

### Sample size

Based on prior research [4], it was estimated that the memory retention rate at 1 h post-surgery would be 50% and 10% in the flumazenil and without flumazenil groups, respectively. Overall, 44 patients were required (22 per group) using a chi-square test with a two-sided significance level of 0.05, 80% power, and an assumed 10% dropout rate.

### Randomization

After the principal investigator enrolled patients in the INDICE cloud system (https://www.umin.ac.jp/indice/cloud.html), participants were randomly assigned to one of two groups in a 1:1 ratio: flumazenil or no flumazenil. This open-label study involved no blinded allocation. Random assignment followed a permuted block design with random block sizes of two and four.

### Statistical analysis

Data for continuous variables were presented as means (standard deviations) or medians (interquartile ranges), whereas categorical data were expressed as percentages. Differences in secondary outcomes between the groups were assessed using appropriate statistical tests as follows: continuous variables were analyzed using Student’s *t*-test, while categorical variables were analyzed using a chi-square test or Fisher’s exact test. All analyses used a two-sided significance level of 0.05.

## Results

### Baseline characteristics

**Fig. 2:**
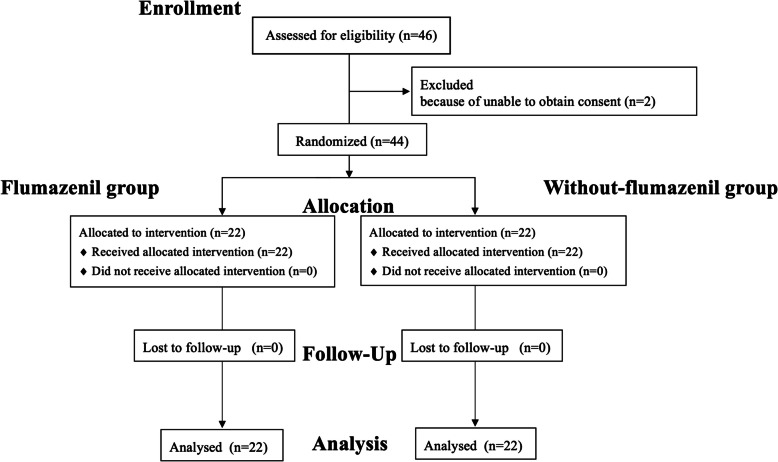
Flow diagram

Overall, 46 patients scheduled for breast surgery at Kyushu University Hospital between November 2023 and July 2024 were screened for eligibility. Two patients were excluded before randomization due to withdrawal of consent, leaving 44 patients for analysis (22 in the without flumazenil group and 22 in the with flumazenil group) (Fig. 2). Memory retention data were complete for all patients, and no imputation was necessary. One patient in each group was taking psychotropic medication. No patients received premedication.

Preoperative and intraoperative characteristics are presented in Table 1. No significant difference was observed. Additionally, no significant differences were noted between the two groups regarding analgesic and antiemetic use postoperatively (Table 2). On average, images related to memory were presented 9 min after flumazenil administration.

**Table 1:** Basic characteristics

	Flumazenil (+)	Flumazenil (−)	*p* value*
	(*n* = 22)	(*n* = 22)	
**Preoperative characteristic values**			
Age, years	49.6 (9.4)	47.5 (9.4)	0.46
Height, cm	157.4 (6.7)	159.5 (4.8)	0.25
Weight, kg	55.6 (9.4)	55.6 (10.2)	0.99
ASA physical status, number			
I/II/III	4/18/0	9/12/1	0.10
Oral hypnotic drugs on the day of surgery, %	4.6	4.6	1.00^†^
**Intraoperative characteristic values**			
Operating time, min	105 (86–144)	92 (74–119)	0.17
Anesthesia time, min	156 (131–219)	142 (120–175)	0.15
Total dose of remimazolam, mg	101 (72–143)	88 (76–114)	0.68
Total dose of fentanyl, μg/kg	4.4 (3.7–5.3)	3.5 (2.8–4.2)	0.02
ESC of remimazolam at extubation, µg/mL	0.31 (0.08)	0.31(0.08)	0.87
ESC of Fentanyl at extubation, ng/mL	0.84 (0.31)	0.76 (0.35)	0.43
Elapsed time until extubation from terminating the infusion of remimazolam, min	12 (11–14)	13 (11–17)	0.36

^*^p-values are based on the unpaired *t*-test or chi-square test, unless otherwise specified

^†^Fisher’s exact test values are presented as means (standard deviation (SD)), medians (interquartile range (IQR)), or percentages

Values are represented as means (standard deviations (SD)), median (interquartile range (IQR)), and numbers or percentages

*ASA* American Society of Anesthesiologists, *BIS* bispectrality index, *ESC* effect-site concentration, *OR* operating room, *SD* standard deviation

**Table 2:** Comparison of primary and secondary outcomes between the with and without flumazenil groups

	Flumazenil (+)	Flumazenil (−)	*p* value*
	(*n* = 22)	(*n* = 22)	
**Primary outcome**			
Remembering the poster 1 h after leaving the OR %	95.5	40.9	< 0.001
Remembering the poster 2 h after leaving the OR %	100	100	
**Secondary outcome**			
Use of analgesics, %	31.8	31.8	1.00^†^
Use of antiemetics, %	22.7	9.1	0.41^†^

^*^p-values are based on the unpaired *t*-test or chi-square test, unless otherwise specified. †Fisher’s exact test values are presented as means (standard deviation (SD)), medians (interquartile range (IQR)), or percentages

*IQR* interquartile range, *OR* operating room

### Recovery of memory following recovery of consciousness

The percentage of patients who recalled the poster 1 h after leaving the operating room (OR) was significantly higher in the flumazenil group than in the without flumazenil group (95.5 vs 40.9%, *p* < 0.001). All patients recalled the poster 2 h after leaving the OR (Table 2). All patients in both groups successfully memorized the poster until 2 h postoperatively.

### The effect-site concentration

The mean effect-site concentration of remimazolam at the time of consciousness recovery was similar between the two groups (0.31 ± 0.08 µg/mL in both groups, *p* = 0.87, Table 1). Furthermore, no statistically significant difference was observed in the mean effect-site concentration of fentanyl at the time of consciousness recovery between the two groups (0.84 ± 0.31 vs. 0.76 ± 0.35 ng/mL, *p* = 0.43, Table 1). The mean effect-site concentration of remimazolam at 1 h after leaving the OR was also similar between the two groups (0.07 ± 0.02 µg/mL in both groups, *p* = 0.88).

No adverse or unanticipated effects occurred in each group.

## Discussion

This study demonstrated that flumazenil significantly accelerated the recovery of memory (memory retention) in patients regaining consciousness after general anesthesia with remimazolam. The clinical significance is that even if patients respond to verbal stimuli after extubation, they may still experience impaired memory retention. However, administering flumazenil is likely to enable patients to retain their memory immediately after extubation.

To compare the time required for the recovery of memory, this study used the same point at which consciousness was regained in response to verbal stimuli as the baseline. To ensure equivalent levels of consciousness, the modified Aldrete score was also used.

Memory involves the following three processes: recognition, retention, and recall. When patients were shown the poster and could accurately describe its content, it indicated that their memory recognition was intact.

This suggests that the difference in recovery of memory between the two groups was due to memory retention or recall. However, no difference was observed in blood concentration 1 h after leaving the OR between the two groups. Therefore, we believe that this study’s results were due to the effect of flumazenil on memory retention. Importantly, the data also revealed that by 2 h postoperatively, all patients, regardless of group, had recovered their ability to recognize, retain, and recall memory, with significant recovery evident as early as 1 h after leaving the OR. However, further research is needed because we could not examine the factors that affect retention.

These results are clinically significant not only after general anesthesia but also in the care setting following procedures such as outpatient surgery and endoscopic examinations under sedation with remimazolam. Generally, when remimazolam is used in day surgery or an endoscopic procedure, lower concentrations than those in this study have been employed. For example, in colonoscopy and esophagogastroduodenoscopy, the total dose of required remimazolam to obtain adequate sedation was 10.2 ± 6.2 and 13.2 ± 8.7 mg, respectively [10]. In a state where patients are conscious but have no memory, there is a possibility that they may engage in socially problematic behavior, which could lead to serious social issues. Our results suggest the duration of observation required after using remimazolam to avoid this risk. Patients who regain consciousness after remimazolam administration are likely to recover memory within 2 h after the start of observation and may be discharged from the hospital.

However, the routine flumazenil administration to all patients receiving remimazolam remains a subject of debate. Routine use of flumazenil should be avoided because using antagonists at emergence may be associated with re-sedation risk [11]. Flumazenil can induce seizures and withdrawal symptoms in patients who have long-term benzodiazepine exposure or have developed tolerance or dependence [12]. Generally, flumazenil is used to promote the recovery of consciousness. Interestingly, this study’s results confirmed that administering flumazenil to patients who had regained consciousness after general anesthesia also accelerated the recovery of memory. Furthermore, the duration of action of flumazenil, with a half-life of 49–52 min, closely aligns with the elimination half-life of remimazolam [13], suggesting that the risk of recurrence of hypnotic effects or memory impairment after the effects of flumazenil have worn off is low, making it clinically useful. However, when assessing psychomotor recovery using the Trieger dot test 30–120 min post-surgery, remimazolam demonstrated delayed psychomotor recovery even with flumazenil, compared to propofol [13]. Although the assessment methods were different, this study showed that the effect site concentration of remimazolam resulted in significant recovery of memory 1 h after leaving the OR. Importantly, the ability to recognize, retain, and recall memories was recovered within 2 h after recovery of consciousness with or without flumazenil administration. If memory recovery is expected earlier than this, it is recommended to use flumazenil after regaining consciousness. However, the overall recovery time for memory retention remains a valuable clinical insight. The finding of 100% memory retention at 1 h, with or without flumazenil administration, suggests that 1 h of postoperative rest is sufficient for patients at even lower doses.

### Limitations

This study had some limitations. First, it was conducted at a single center, which may limit the generalizability of the findings. Second, the study included only female and younger patients, which may introduce a gender and age bias. However, no significant differences in memory retention between genders have been reported in previous studies [14]. Additionally, older adult patients, males, and those undergoing other surgeries may have different results. Third, re-sedation has not been assessed. Finally, this study was conducted as an open-label trial. The anesthesiologist in charge was aware of whether or not flumazenil had been administered to the patients; however, the patients were not informed of the group they had been assigned to. While the effect on the results was not zero, we considered that the lack of double-blinding for patients did not significantly affect the results, as they were evaluated by a third party. Conversely, patients were informed that their memory would be assessed when obtaining study consent. This might have influenced the results because the patients consciously attempted to remember the posters and concentrated more than usual.

## Conclusions

Flumazenil significantly accelerated the recovery of memory retention in patients after general anesthesia with remimazolam. However, in this study, all patients in both the flumazenil and without flumazenil groups successfully recovered their memory until 2 h after regaining consciousness.

## Supplementary Information

Additional file 1

## Data Availability

Not applicable.

## Abbreviations

PSI: Patient state index
OR: Operating room
