# Supplementary figures and images for: Effect of flumazenil on recovery of memory following recovery of consciousness from general anesthesia with remimazolam: a randomized, open-label, single-center controlled trial
Source: JA Clin Rep. 2025 Oct 21;11:58. doi: 10.1186/s40981-025-00825-5 (PMC12540950; doi:10.1186/s40981-025-00825-5)

**Supplement 1**

Illustration A


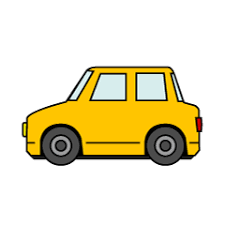


Illustration B


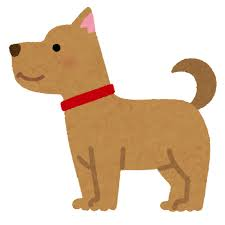


Illustration C


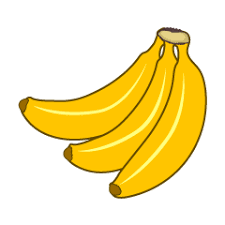

Supplement: Supplementary file 1 — Additional file 1 [file 40981_2025_825_MOESM1_ESM.docx]
